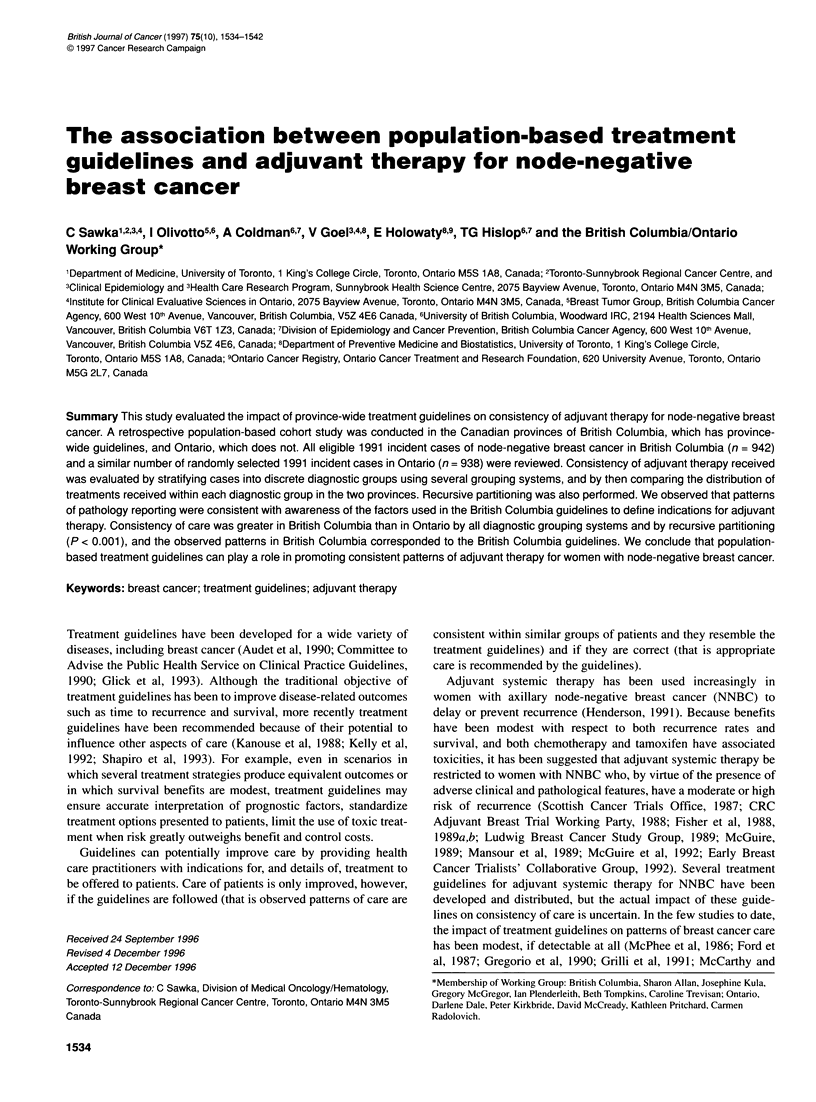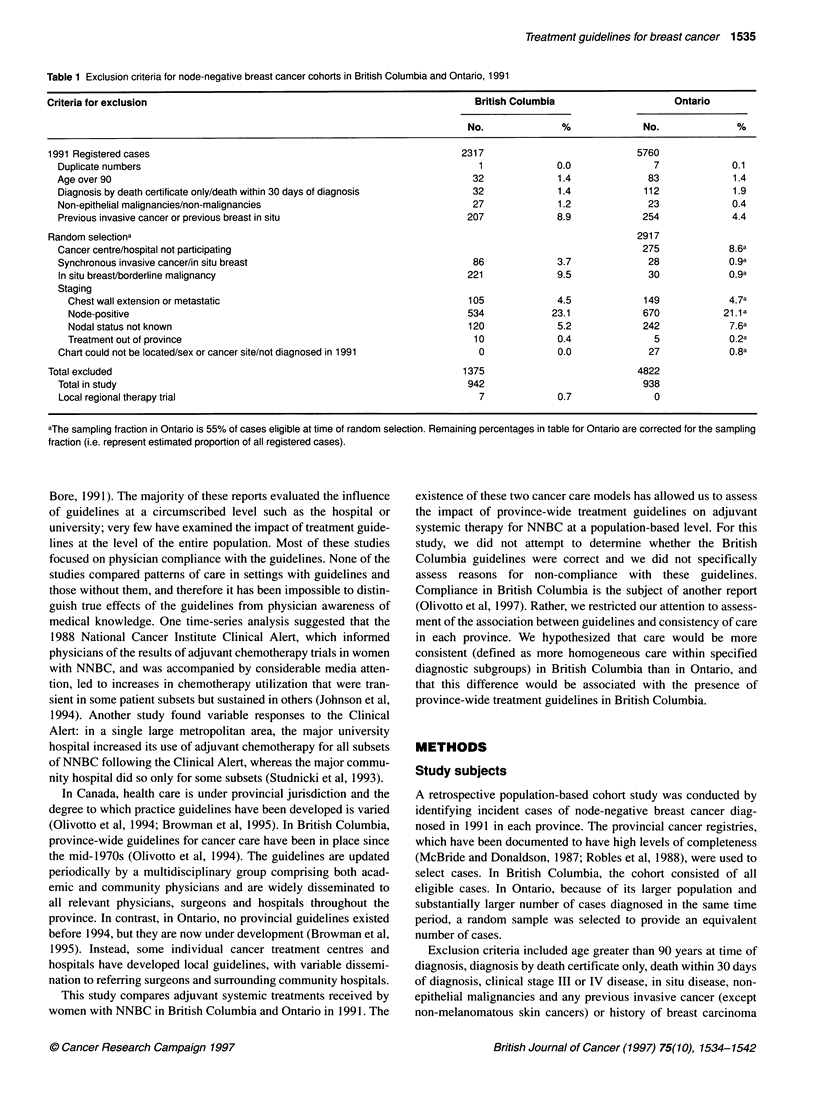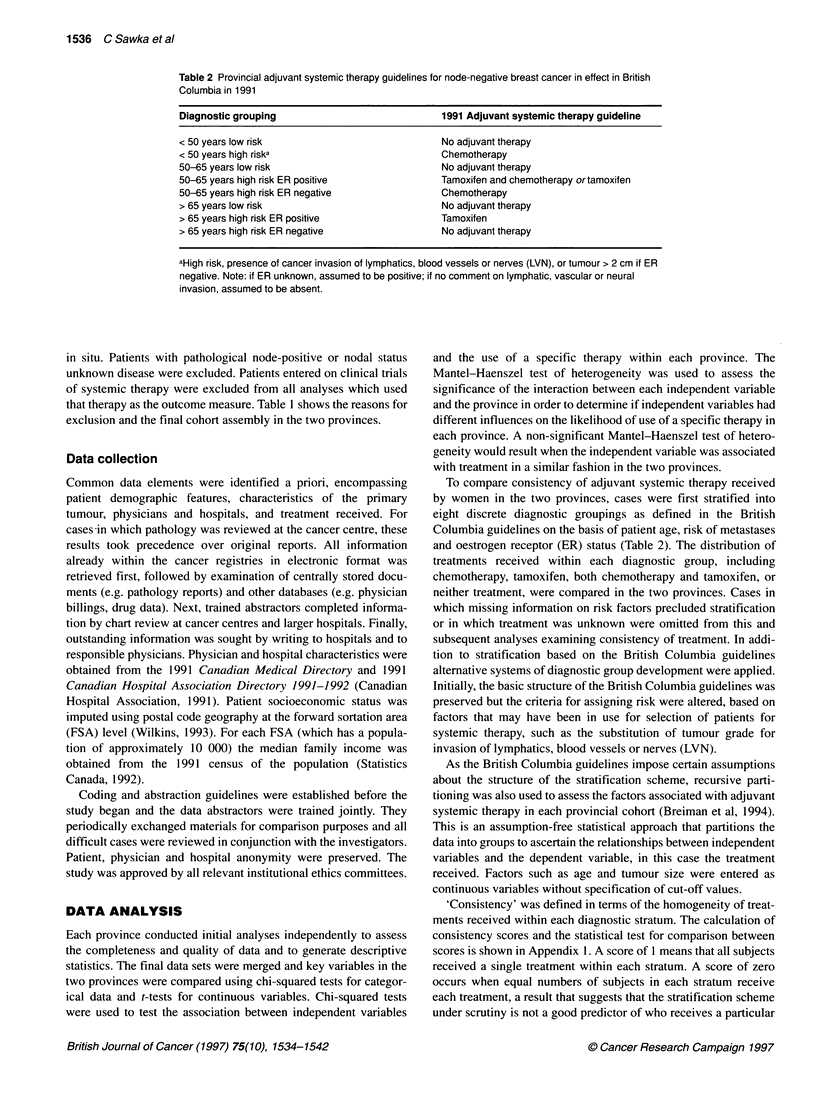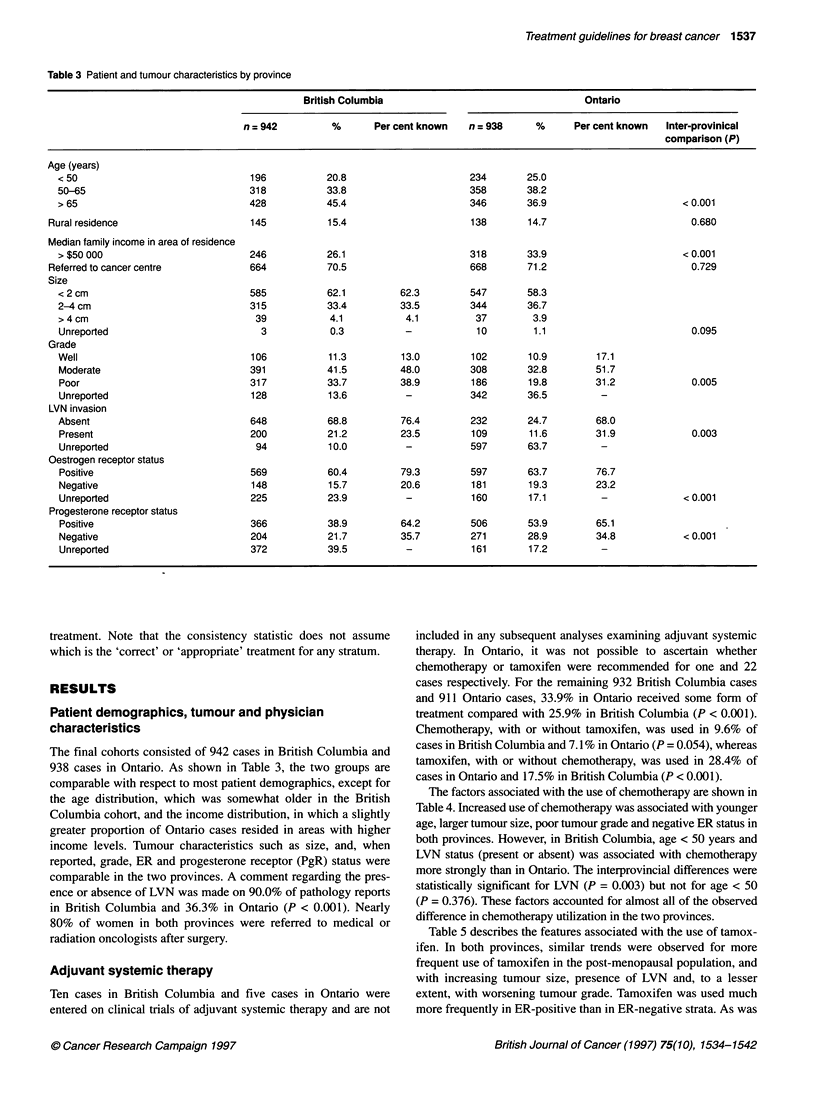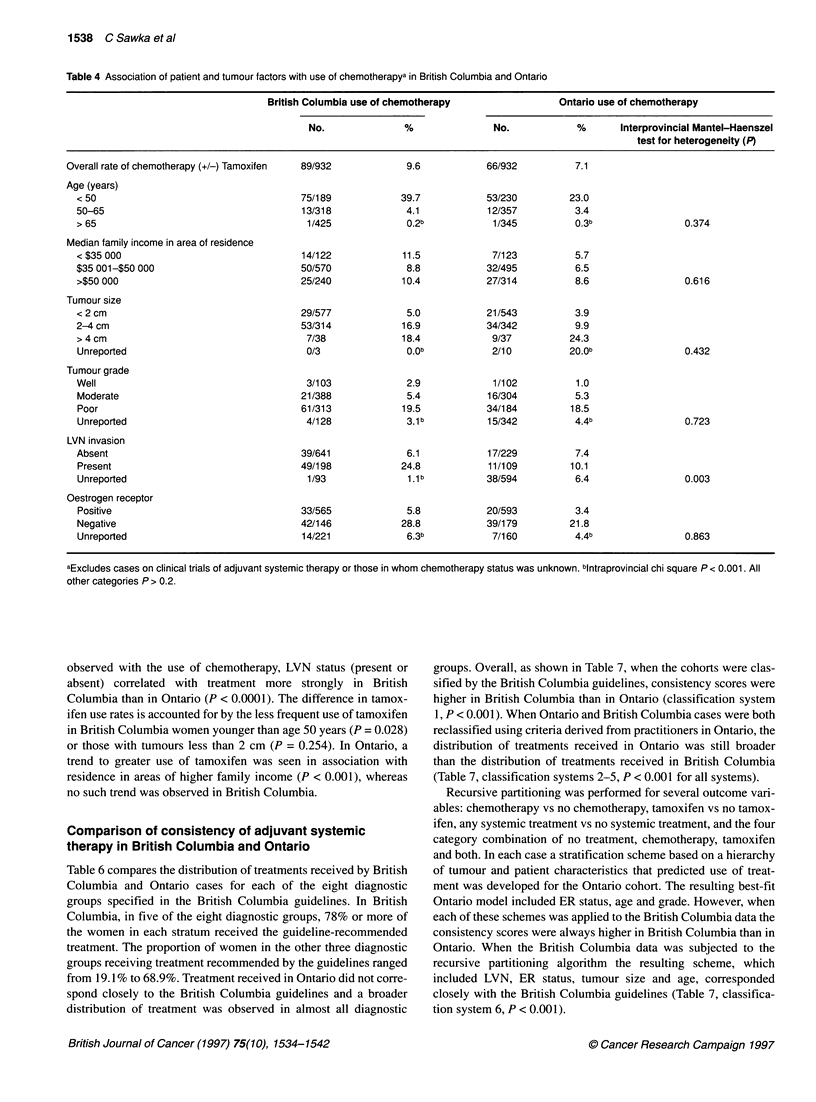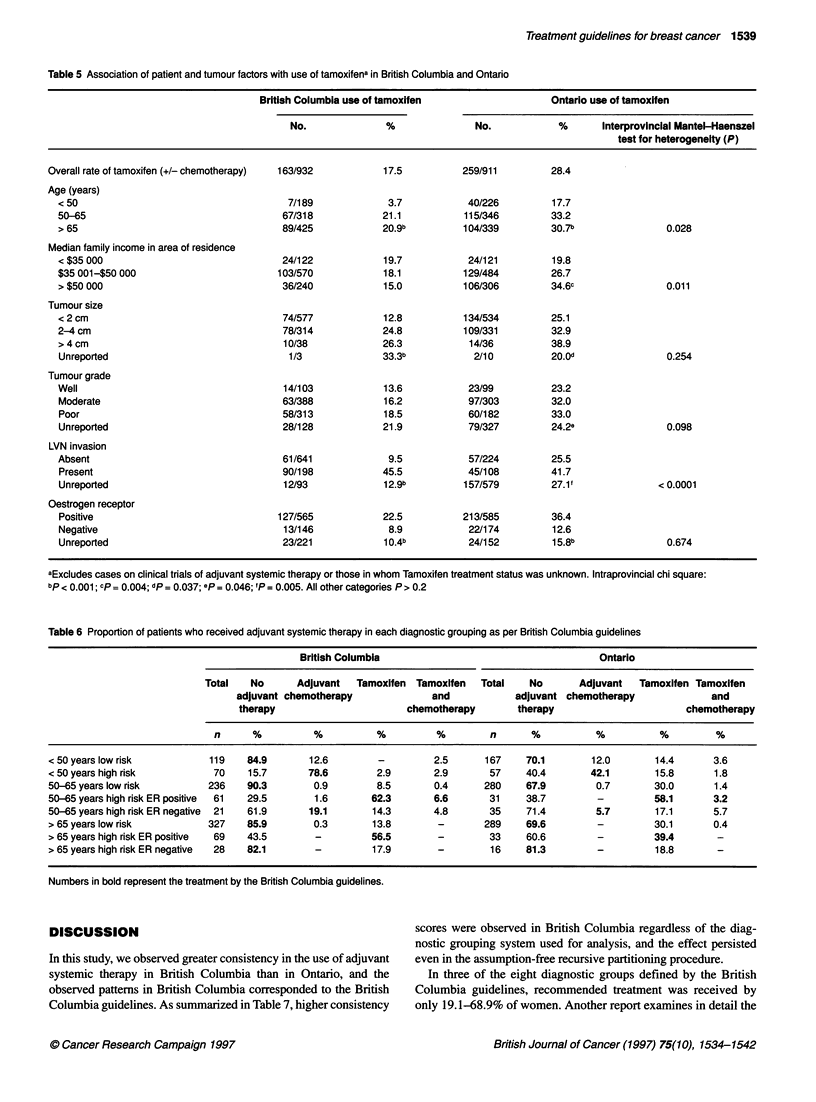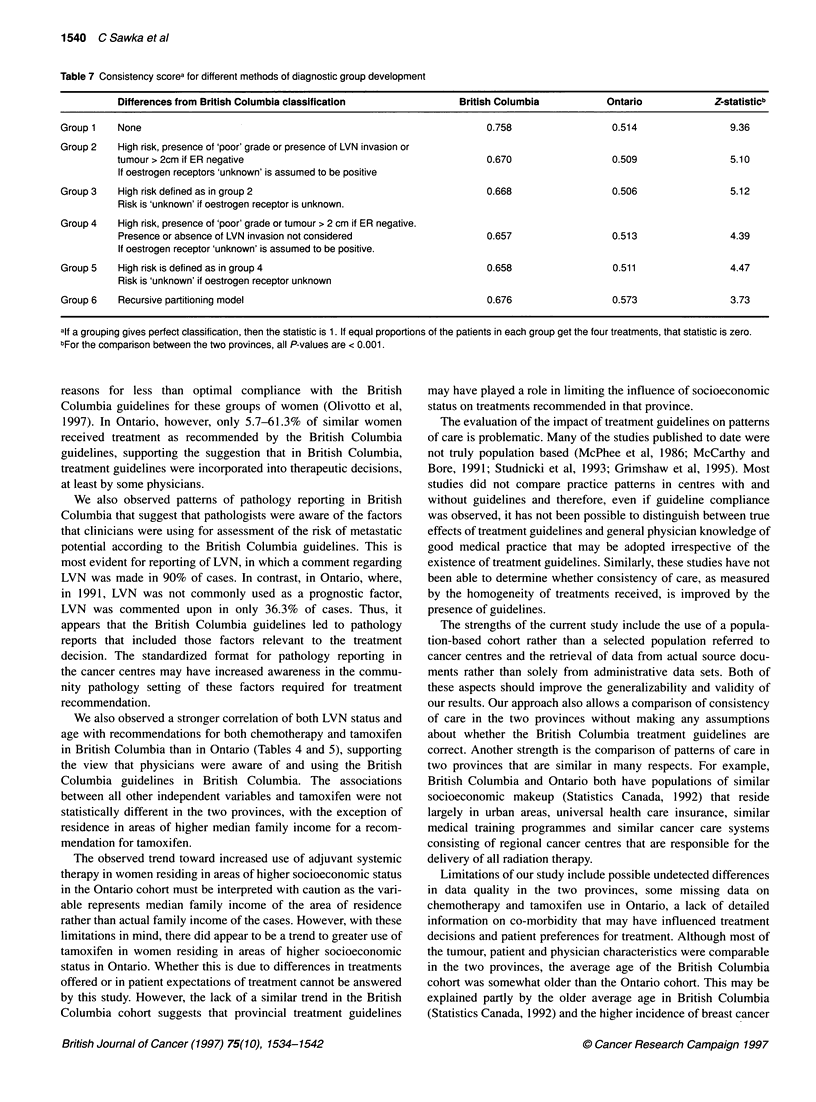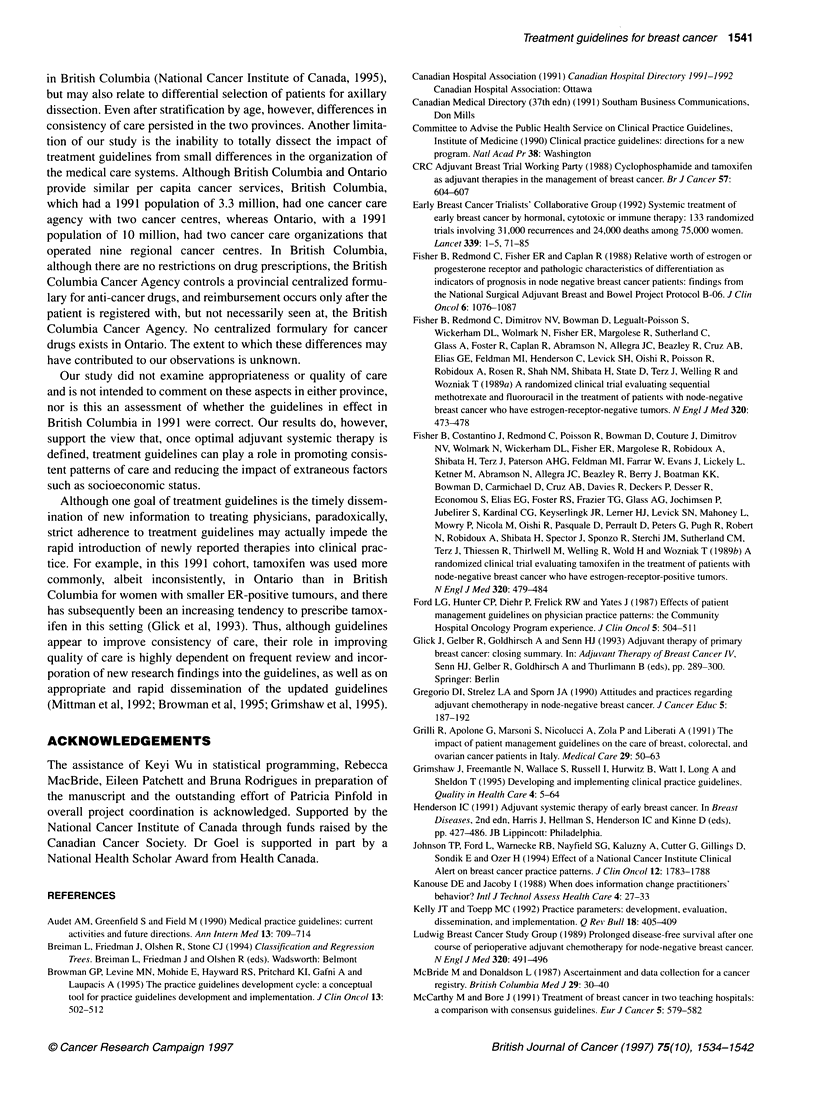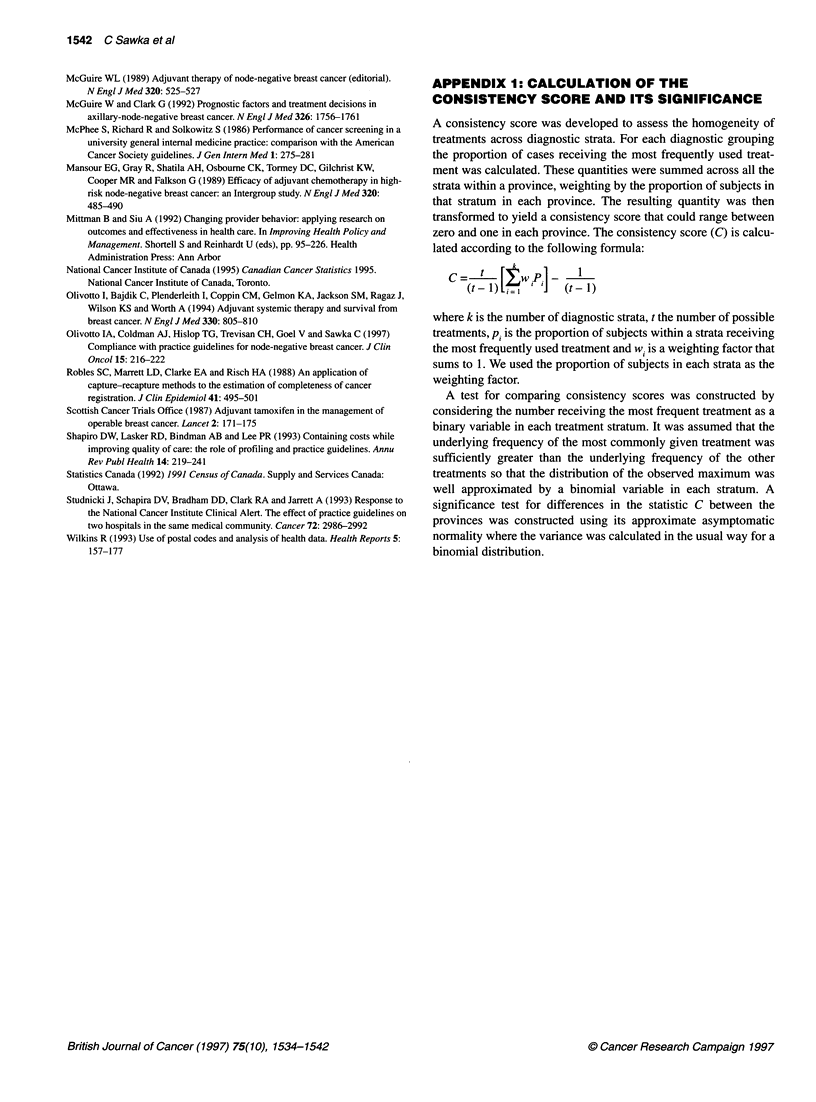# The association between population-based treatment guidelines and adjuvant therapy for node-negative breast cancer. British Columbia/Ontario Working Group.

**DOI:** 10.1038/bjc.1997.262

**Published:** 1997

**Authors:** C. Sawka, I. Olivotto, A. Coldman, V. Goel, E. Holowaty, T. G. Hislop

**Affiliations:** Department of Medicine, University of Toronto, Ontario, Canada.

## Abstract

This study evaluated the impact of province-wide treatment guidelines on consistency of adjuvant therapy for node-negative breast cancer. A retrospective population-based cohort study was conducted in the Canadian provinces of British Columbia, which has province-wide guidelines, and Ontario, which does not. All eligible 1991 incident cases of node-negative breast cancer in British Columbia (n = 942) and a similar number of randomly selected 1991 incident cases in Ontario (n = 938) were reviewed. Consistency of adjuvant therapy received was evaluated by stratifying cases into discrete diagnostic groups using several grouping systems, and by then comparing the distribution of treatments received within each diagnostic group in the two provinces. Recursive partitioning was also performed. We observed that patterns of pathology reporting were consistent with awareness of the factors used in the British Columbia guidelines to define indications for adjuvant therapy. Consistency of care was greater in British Columbia than in Ontario by all diagnostic grouping systems and by recursive partitioning (P < 0.001), and the observed patterns in British Columbia corresponded to the British Columbia guidelines. We conclude that population-based treatment guidelines can play a role in promoting consistent patterns of adjuvant therapy for women with node-negative breast cancer.


British Journal of Cancer (1997) 75(10), 1534–1542
© 1997 Cancer Research Campaign

### The association between population-based treatment guidelines and adjuvant therapy for node-negative breast cancer

**C Sawka[1,2,3,4], I Olivotto[5,6], A Coldman[6,7], V Goel[3,4,8], E Holowaty[8,9], TG Hislop[6,7] and the British Columbia/Ontario Working Group***

[1]Department of Medicine, University of Toronto, 1 King's College Circle, Toronto, Ontario M5S 1A8, Canada; [2]Toronto-Sunnybrook Regional Cancer Centre, and [3]Clinical Epidemiology and [3]Health Care Research Program, Sunnybrook Health Science Centre, 2075 Bayview Avenue, Toronto, Ontario M4N 3M5, Canada; [4]Institute for Clinical Evaluative Sciences in Ontario, 2075 Bayview Avenue, Toronto, Ontario M4N 3M5, Canada, [5]Breast Tumor Group, British Columbia Cancer Agency, 600 West 10th Avenue, Vancouver, British Columbia, V5Z 4E6 Canada, [6]University of British Columbia, Woodward IRC, 2194 Health Sciences Mall, Vancouver, British Columbia V6T 1Z3, Canada; [7]Division of Epidemiology and Cancer Prevention, British Columbia Cancer Agency, 600 West 10th Avenue, Vancouver, British Columbia V5Z 4E6, Canada; [8]Department of Preventive Medicine and Biostatistics, University of Toronto, 1 King's College Circle, Toronto, Ontario M5S 1A8, Canada; [9]Ontario Cancer Registry, Ontario Cancer Treatment and Research Foundation, 620 University Avenue, Toronto, Ontario M5G 2L7, Canada

**Summary** This study evaluated the impact of province-wide treatment guidelines on consistency of adjuvant therapy for node-negative breast cancer. A retrospective population-based cohort study was conducted in the Canadian provinces of British Columbia, which has province-wide guidelines, and Ontario, which does not. All eligible 1991 incident cases of node-negative breast cancer in British Columbia ($n$ = 942) and a similar number of randomly selected 1991 incident cases in Ontario ($n$ = 938) were reviewed. Consistency of adjuvant therapy received was evaluated by stratifying cases into discrete diagnostic groups using several grouping systems, and by then comparing the distribution of treatments received within each diagnostic group in the two provinces. Recursive partitioning was also performed. We observed that patterns of pathology reporting were consistent with awareness of the factors used in the British Columbia guidelines to define indications for adjuvant therapy. Consistency of care was greater in British Columbia than in Ontario by all diagnostic grouping systems and by recursive partitioning ($P$ < 0.001), and the observed patterns in British Columbia corresponded to the British Columbia guidelines. We conclude that population-based treatment guidelines can play a role in promoting consistent patterns of adjuvant therapy for women with node-negative breast cancer.

**Keywords:** breast cancer; treatment guidelines; adjuvant therapy

Treatment guidelines have been developed for a wide variety of diseases, including breast cancer (Audet et al, 1990; Committee to Advise the Public Health Service on Clinical Practice Guidelines, 1990; Glick et al, 1993). Although the traditional objective of treatment guidelines has been to improve disease-related outcomes such as time to recurrence and survival, more recently treatment guidelines have been recommended because of their potential to influence other aspects of care (Kanouse et al, 1988; Kelly et al, 1992; Shapiro et al, 1993). For example, even in scenarios in which several treatment strategies produce equivalent outcomes or in which survival benefits are modest, treatment guidelines may ensure accurate interpretation of prognostic factors, standardize treatment options presented to patients, limit the use of toxic treatment when risk greatly outweighs benefit and control costs.

Guidelines can potentially improve care by providing health care practitioners with indications for, and details of, treatment to be offered to patients. Care of patients is only improved, however, if the guidelines are followed (that is observed patterns of care are consistent within similar groups of patients and they resemble the treatment guidelines) and if they are correct (that is appropriate care is recommended by the guidelines).

Adjuvant systemic therapy has been used increasingly in women with axillary node-negative breast cancer (NNBC) to delay or prevent recurrence (Henderson, 1991). Because benefits have been modest with respect to both recurrence rates and survival, and both chemotherapy and tamoxifen have associated toxicities, it has been suggested that adjuvant systemic therapy be restricted to women with NNBC who, by virtue of the presence of adverse clinical and pathological features, have a moderate or high risk of recurrence (Scottish Cancer Trials Office, 1987; CRC Adjuvant Breast Trial Working Party, 1988; Fisher et al, 1988, 1989*a,b*; Ludwig Breast Cancer Study Group, 1989; McGuire, 1989; Mansour et al, 1989; McGuire et al, 1992; Early Breast Cancer Trialists' Collaborative Group, 1992). Several treatment guidelines for adjuvant systemic therapy for NNBC have been developed and distributed, but the actual impact of these guidelines on consistency of care is uncertain. In the few studies to date, the impact of treatment guidelines on patterns of breast cancer care has been modest, if detectable at all (McPhee et al, 1986; Ford et al, 1987; Gregorio et al, 1990; Grilli et al, 1991; McCarthy and

*Received 24 September 1996*
*Revised 4 December 1996*
*Accepted 12 December 1996*

*Correspondence to:* C Sawka, Division of Medical Oncology/Hematology, Toronto-Sunnybrook Regional Cancer Centre, Toronto, Ontario M4N 3M5 Canada

*Membership of Working Group: British Columbia, Sharon Allan, Josephine Kula, Gregory McGregor, Ian Plenderleith, Beth Tompkins, Caroline Trevisan; Ontario, Darlene Dale, Peter Kirkbride, David McCready, Kathleen Pritchard, Carmen Radolovich.

**Table 1** Exclusion criteria for node-negative breast cancer cohorts in British Columbia and Ontario, 1991

| Criteria for exclusion | British Columbia | | Ontario | |
|---|---|---|---|---|
| | No. | % | No. | % |
| 1991 Registered cases | 2317 | | 5760 | |
| Duplicate numbers | 1 | 0.0 | 7 | 0.1 |
| Age over 90 | 32 | 1.4 | 83 | 1.4 |
| Diagnosis by death certificate only/death within 30 days of diagnosis | 32 | 1.4 | 112 | 1.9 |
| Non-epithelial malignancies/non-malignancies | 27 | 1.2 | 23 | 0.4 |
| Previous invasive cancer or previous breast in situ | 207 | 8.9 | 254 | 4.4 |
| Random selection[a] | | | 2917 | |
| Cancer centre/hospital not participating | | | 275 | 8.6[a] |
| Synchronous invasive cancer/in situ breast | 86 | 3.7 | 28 | 0.9[a] |
| In situ breast/borderline malignancy | 221 | 9.5 | 30 | 0.9[a] |
| Staging | | | | |
| Chest wall extension or metastatic | 105 | 4.5 | 149 | 4.7[a] |
| Node-positive | 534 | 23.1 | 670 | 21.1[a] |
| Nodal status not known | 120 | 5.2 | 242 | 7.6[a] |
| Treatment out of province | 10 | 0.4 | 5 | 0.2[a] |
| Chart could not be located/sex or cancer site/not diagnosed in 1991 | 0 | 0.0 | 27 | 0.8[a] |
| Total excluded | 1375 | | 4822 | |
| Total in study | 942 | | 938 | |
| Local regional therapy trial | 7 | 0.7 | 0 | |

[a]The sampling fraction in Ontario is 55% of cases eligible at time of random selection. Remaining percentages in table for Ontario are corrected for the sampling fraction (i.e. represent estimated proportion of all registered cases).

Bore, 1991). The majority of these reports evaluated the influence of guidelines at a circumscribed level such as the hospital or university; very few have examined the impact of treatment guidelines at the level of the entire population. Most of these studies focused on physician compliance with the guidelines. None of the studies compared patterns of care in settings with guidelines and those without them, and therefore it has been impossible to distinguish true effects of the guidelines from physician awareness of medical knowledge. One time-series analysis suggested that the 1988 National Cancer Institute Clinical Alert, which informed physicians of the results of adjuvant chemotherapy trials in women with NNBC, and was accompanied by considerable media attention, led to increases in chemotherapy utilization that were transient in some patient subsets but sustained in others (Johnson et al, 1994). Another study found variable responses to the Clinical Alert: in a single large metropolitan area, the major university hospital increased its use of adjuvant chemotherapy for all subsets of NNBC following the Clinical Alert, whereas the major community hospital did so only for some subsets (Studnicki et al, 1993).

In Canada, health care is under provincial jurisdiction and the degree to which practice guidelines have been developed is varied (Olivotto et al, 1994; Browman et al, 1995). In British Columbia, province-wide guidelines for cancer care have been in place since the mid-1970s (Olivotto et al, 1994). The guidelines are updated periodically by a multidisciplinary group comprising both academic and community physicians and are widely disseminated to all relevant physicians, surgeons and hospitals throughout the province. In contrast, in Ontario, no provincial guidelines existed before 1994, but they are now under development (Browman et al, 1995). Instead, some individual cancer treatment centres and hospitals have developed local guidelines, with variable dissemination to referring surgeons and surrounding community hospitals.

This study compares adjuvant systemic treatments received by women with NNBC in British Columbia and Ontario in 1991. The existence of these two cancer care models has allowed us to assess the impact of province-wide treatment guidelines on adjuvant systemic therapy for NNBC at a population-based level. For this study, we did not attempt to determine whether the British Columbia guidelines were correct and we did not specifically assess reasons for non-compliance with these guidelines. Compliance in British Columbia is the subject of another report (Olivotto et al, 1997). Rather, we restricted our attention to assessment of the association between guidelines and consistency of care in each province. We hypothesized that care would be more consistent (defined as more homogeneous care within specified diagnostic subgroups) in British Columbia than in Ontario, and that this difference would be associated with the presence of province-wide treatment guidelines in British Columbia.

### METHODS

### Study subjects

A retrospective population-based cohort study was conducted by identifying incident cases of node-negative breast cancer diagnosed in 1991 in each province. The provincial cancer registries, which have been documented to have high levels of completeness (McBride and Donaldson, 1987; Robles et al, 1988), were used to select cases. In British Columbia, the cohort consisted of all eligible cases. In Ontario, because of its larger population and substantially larger number of cases diagnosed in the same time period, a random sample was selected to provide an equivalent number of cases.

Exclusion criteria included age greater than 90 years at time of diagnosis, diagnosis by death certificate only, death within 30 days of diagnosis, clinical stage III or IV disease, in situ disease, non-epithelial malignancies and any previous invasive cancer (except non-melanomatous skin cancers) or history of breast carcinoma

© Cancer Research Campaign 1997

Table 2 Provincial adjuvant systemic therapy guidelines for node-negative breast cancer in effect in British Columbia in 1991

| Diagnostic grouping | 1991 Adjuvant systemic therapy guideline |
|---|---|
| < 50 years low risk | No adjuvant therapy |
| < 50 years high risk[a] | Chemotherapy |
| 50–65 years low risk | No adjuvant therapy |
| 50–65 years high risk ER positive | Tamoxifen and chemotherapy *or* tamoxifen |
| 50–65 years high risk ER negative | Chemotherapy |
| > 65 years low risk | No adjuvant therapy |
| > 65 years high risk ER positive | Tamoxifen |
| > 65 years high risk ER negative | No adjuvant therapy |

[a]High risk, presence of cancer invasion of lymphatics, blood vessels or nerves (LVN), or tumour > 2 cm if ER negative. Note: if ER unknown, assumed to be positive; if no comment on lymphatic, vascular or neural invasion, assumed to be absent.

in situ. Patients with pathological node-positive or nodal status unknown disease were excluded. Patients entered on clinical trials of systemic therapy were excluded from all analyses which used that therapy as the outcome measure. Table 1 shows the reasons for exclusion and the final cohort assembly in the two provinces.

### Data collection

Common data elements were identified a priori, encompassing patient demographic features, characteristics of the primary tumour, physicians and hospitals, and treatment received. For cases in which pathology was reviewed at the cancer centre, these results took precedence over original reports. All information already within the cancer registries in electronic format was retrieved first, followed by examination of centrally stored documents (e.g. pathology reports) and other databases (e.g. physician billings, drug data). Next, trained abstractors completed information by chart review at cancer centres and larger hospitals. Finally, outstanding information was sought by writing to hospitals and to responsible physicians. Physician and hospital characteristics were obtained from the 1991 *Canadian Medical Directory* and 1991 *Canadian Hospital Association Directory 1991–1992* (Canadian Hospital Association, 1991). Patient socioeconomic status was imputed using postal code geography at the forward sortation area (FSA) level (Wilkins, 1993). For each FSA (which has a population of approximately 10 000) the median family income was obtained from the 1991 census of the population (Statistics Canada, 1992).

Coding and abstraction guidelines were established before the study began and the data abstractors were trained jointly. They periodically exchanged materials for comparison purposes and all difficult cases were reviewed in conjunction with the investigators. Patient, physician and hospital anonymity were preserved. The study was approved by all relevant institutional ethics committees.

### DATA ANALYSIS

Each province conducted initial analyses independently to assess the completeness and quality of data and to generate descriptive statistics. The final data sets were merged and key variables in the two provinces were compared using chi-squared tests for categorical data and *t*-tests for continuous variables. Chi-squared tests were used to test the association between independent variables and the use of a specific therapy within each province. The Mantel–Haenszel test of heterogeneity was used to assess the significance of the interaction between each independent variable and the province in order to determine if independent variables had different influences on the likelihood of use of a specific therapy in each province. A non-significant Mantel–Haenszel test of heterogeneity would result when the independent variable was associated with treatment in a similar fashion in the two provinces.

To compare consistency of adjuvant systemic therapy received by women in the two provinces, cases were first stratified into eight discrete diagnostic groupings as defined in the British Columbia guidelines on the basis of patient age, risk of metastases and oestrogen receptor (ER) status (Table 2). The distribution of treatments received within each diagnostic group, including chemotherapy, tamoxifen, both chemotherapy and tamoxifen, or neither treatment, were compared in the two provinces. Cases in which missing information on risk factors precluded stratification or in which treatment was unknown were omitted from this and subsequent analyses examining consistency of treatment. In addition to stratification based on the British Columbia guidelines alternative systems of diagnostic group development were applied. Initially, the basic structure of the British Columbia guidelines was preserved but the criteria for assigning risk were altered, based on factors that may have been in use for selection of patients for systemic therapy, such as the substitution of tumour grade for invasion of lymphatics, blood vessels or nerves (LVN).

As the British Columbia guidelines impose certain assumptions about the structure of the stratification scheme, recursive partitioning was also used to assess the factors associated with adjuvant systemic therapy in each provincial cohort (Breiman et al, 1994). This is an assumption-free statistical approach that partitions the data into groups to ascertain the relationships between independent variables and the dependent variable, in this case the treatment received. Factors such as age and tumour size were entered as continuous variables without specification of cut-off values.

'Consistency' was defined in terms of the homogeneity of treatments received within each diagnostic stratum. The calculation of consistency scores and the statistical test for comparison between scores is shown in Appendix 1. A score of 1 means that all subjects received a single treatment within each stratum. A score of zero occurs when equal numbers of subjects in each stratum receive each treatment, a result that suggests that the stratification scheme under scrutiny is not a good predictor of who receives a particular

 © Cancer Research Campaign 1997

**Table 3** Patient and tumour characteristics by province

| | British Columbia | | | Ontario | | | |
|---|---|---|---|---|---|---|---|
| | *n* = 942 | % | Per cent known | *n* = 938 | % | Per cent known | Inter-provinical comparison (*P*) |
| Age (years) | | | | | | | |
| < 50 | 196 | 20.8 | | 234 | 25.0 | | |
| 50–65 | 318 | 33.8 | | 358 | 38.2 | | |
| > 65 | 428 | 45.4 | | 346 | 36.9 | | < 0.001 |
| Rural residence | 145 | 15.4 | | 138 | 14.7 | | 0.680 |
| Median family income in area of residence | | | | | | | |
| > $50 000 | 246 | 26.1 | | 318 | 33.9 | | < 0.001 |
| Referred to cancer centre | 664 | 70.5 | | 668 | 71.2 | | 0.729 |
| Size | | | | | | | |
| < 2 cm | 585 | 62.1 | 62.3 | 547 | 58.3 | | |
| 2–4 cm | 315 | 33.4 | 33.5 | 344 | 36.7 | | |
| > 4 cm | 39 | 4.1 | 4.1 | 37 | 3.9 | | |
| Unreported | 3 | 0.3 | – | 10 | 1.1 | | 0.095 |
| Grade | | | | | | | |
| Well | 106 | 11.3 | 13.0 | 102 | 10.9 | 17.1 | |
| Moderate | 391 | 41.5 | 48.0 | 308 | 32.8 | 51.7 | |
| Poor | 317 | 33.7 | 38.9 | 186 | 19.8 | 31.2 | 0.005 |
| Unreported | 128 | 13.6 | – | 342 | 36.5 | – | |
| LVN invasion | | | | | | | |
| Absent | 648 | 68.8 | 76.4 | 232 | 24.7 | 68.0 | |
| Present | 200 | 21.2 | 23.5 | 109 | 11.6 | 31.9 | 0.003 |
| Unreported | 94 | 10.0 | – | 597 | 63.7 | – | |
| Oestrogen receptor status | | | | | | | |
| Positive | 569 | 60.4 | 79.3 | 597 | 63.7 | 76.7 | |
| Negative | 148 | 15.7 | 20.6 | 181 | 19.3 | 23.2 | |
| Unreported | 225 | 23.9 | – | 160 | 17.1 | – | < 0.001 |
| Progesterone receptor status | | | | | | | |
| Positive | 366 | 38.9 | 64.2 | 506 | 53.9 | 65.1 | |
| Negative | 204 | 21.7 | 35.7 | 271 | 28.9 | 34.8 | < 0.001 |
| Unreported | 372 | 39.5 | – | 161 | 17.2 | – | |

treatment. Note that the consistency statistic does not assume which is the 'correct' or 'appropriate' treatment for any stratum.

### RESULTS

### Patient demographics, tumour and physician characteristics

The final cohorts consisted of 942 cases in British Columbia and 938 cases in Ontario. As shown in Table 3, the two groups are comparable with respect to most patient demographics, except for the age distribution, which was somewhat older in the British Columbia cohort, and the income distribution, in which a slightly greater proportion of Ontario cases resided in areas with higher income levels. Tumour characteristics such as size, and, when reported, grade, ER and progesterone receptor (PgR) status were comparable in the two provinces. A comment regarding the presence or absence of LVN was made on 90.0% of pathology reports in British Columbia and 36.3% in Ontario ($P < 0.001$). Nearly 80% of women in both provinces were referred to medical or radiation oncologists after surgery.

### Adjuvant systemic therapy

Ten cases in British Columbia and five cases in Ontario were entered on clinical trials of adjuvant systemic therapy and are not included in any subsequent analyses examining adjuvant systemic therapy. In Ontario, it was not possible to ascertain whether chemotherapy or tamoxifen were recommended for one and 22 cases respectively. For the remaining 932 British Columbia cases and 911 Ontario cases, 33.9% in Ontario received some form of treatment compared with 25.9% in British Columbia ($P < 0.001$). Chemotherapy, with or without tamoxifen, was used in 9.6% of cases in British Columbia and 7.1% in Ontario ($P = 0.054$), whereas tamoxifen, with or without chemotherapy, was used in 28.4% of cases in Ontario and 17.5% in British Columbia ($P < 0.001$).

The factors associated with the use of chemotherapy are shown in Table 4. Increased use of chemotherapy was associated with younger age, larger tumour size, poor tumour grade and negative ER status in both provinces. However, in British Columbia, age < 50 years and LVN status (present or absent) was associated with chemotherapy more strongly than in Ontario. The interprovincial differences were statistically significant for LVN ($P = 0.003$) but not for age < 50 ($P = 0.376$). These factors accounted for almost all of the observed difference in chemotherapy utilization in the two provinces.

Table 5 describes the features associated with the use of tamoxifen. In both provinces, similar trends were observed for more frequent use of tamoxifen in the post-menopausal population, and with increasing tumour size, presence of LVN and, to a lesser extent, with worsening tumour grade. Tamoxifen was used much more frequently in ER-positive than in ER-negative strata. As was

© *Cancer Research Campaign 1997*

**Table 4** Association of patient and tumour factors with use of chemotherapy[a] in British Columbia and Ontario

| | British Columbia use of chemotherapy | | Ontario use of chemotherapy | | |
|---|---|---|---|---|---|
| | No. | % | No. | % | Interprovincial Mantel–Haenszel test for heterogeneity (P) |
| Overall rate of chemotherapy (+/–) Tamoxifen | 89/932 | 9.6 | 66/932 | 7.1 | |
| Age (years) | | | | | |
| < 50 | 75/189 | 39.7 | 53/230 | 23.0 | |
| 50–65 | 13/318 | 4.1 | 12/357 | 3.4 | |
| > 65 | 1/425 | 0.2[b] | 1/345 | 0.3[b] | 0.374 |
| Median family income in area of residence | | | | | |
| < $35 000 | 14/122 | 11.5 | 7/123 | 5.7 | |
| $35 001–$50 000 | 50/570 | 8.8 | 32/495 | 6.5 | |
| >$50 000 | 25/240 | 10.4 | 27/314 | 8.6 | 0.616 |
| Tumour size | | | | | |
| < 2 cm | 29/577 | 5.0 | 21/543 | 3.9 | |
| 2–4 cm | 53/314 | 16.9 | 34/342 | 9.9 | |
| > 4 cm | 7/38 | 18.4 | 9/37 | 24.3 | |
| Unreported | 0/3 | 0.0[b] | 2/10 | 20.0[b] | 0.432 |
| Tumour grade | | | | | |
| Well | 3/103 | 2.9 | 1/102 | 1.0 | |
| Moderate | 21/388 | 5.4 | 16/304 | 5.3 | |
| Poor | 61/313 | 19.5 | 34/184 | 18.5 | |
| Unreported | 4/128 | 3.1[b] | 15/342 | 4.4[b] | 0.723 |
| LVN invasion | | | | | |
| Absent | 39/641 | 6.1 | 17/229 | 7.4 | |
| Present | 49/198 | 24.8 | 11/109 | 10.1 | |
| Unreported | 1/93 | 1.1[b] | 38/594 | 6.4 | 0.003 |
| Oestrogen receptor | | | | | |
| Positive | 33/565 | 5.8 | 20/593 | 3.4 | |
| Negative | 42/146 | 28.8 | 39/179 | 21.8 | |
| Unreported | 14/221 | 6.3[b] | 7/160 | 4.4[b] | 0.863 |

[a]Excludes cases on clinical trials of adjuvant systemic therapy or those in whom chemotherapy status was unknown. [b]Intraprovincial chi square $P < 0.001$. All other categories $P > 0.2$.

observed with the use of chemotherapy, LVN status (present or absent) correlated with treatment more strongly in British Columbia than in Ontario ($P < 0.0001$). The difference in tamoxifen use rates is accounted for by the less frequent use of tamoxifen in British Columbia women younger than age 50 years ($P = 0.028$) or those with tumours less than 2 cm ($P = 0.254$). In Ontario, a trend to greater use of tamoxifen was seen in association with residence in areas of higher family income ($P < 0.001$), whereas no such trend was observed in British Columbia.

### Comparison of consistency of adjuvant systemic therapy in British Columbia and Ontario

Table 6 compares the distribution of treatments received by British Columbia and Ontario cases for each of the eight diagnostic groups specified in the British Columbia guidelines. In British Columbia, in five of the eight diagnostic groups, 78% or more of the women in each stratum received the guideline-recommended treatment. The proportion of women in the other three diagnostic groups receiving treatment recommended by the guidelines ranged from 19.1% to 68.9%. Treatment received in Ontario did not correspond closely to the British Columbia guidelines and a broader distribution of treatment was observed in almost all diagnostic groups. Overall, as shown in Table 7, when the cohorts were classified by the British Columbia guidelines, consistency scores were higher in British Columbia than in Ontario (classification system 1, $P < 0.001$). When Ontario and British Columbia cases were both reclassified using criteria derived from practitioners in Ontario, the distribution of treatments received in Ontario was still broader than the distribution of treatments received in British Columbia (Table 7, classification systems 2–5, $P < 0.001$ for all systems).

Recursive partitioning was performed for several outcome variables: chemotherapy vs no chemotherapy, tamoxifen vs no tamoxifen, any systemic treatment vs no systemic treatment, and the four category combination of no treatment, chemotherapy, tamoxifen and both. In each case a stratification scheme based on a hierarchy of tumour and patient characteristics that predicted use of treatment was developed for the Ontario cohort. The resulting best-fit Ontario model included ER status, age and grade. However, when each of these schemes was applied to the British Columbia data the consistency scores were always higher in British Columbia than in Ontario. When the British Columbia data was subjected to the recursive partitioning algorithm the resulting scheme, which included LVN, ER status, tumour size and age, corresponded closely with the British Columbia guidelines (Table 7, classification system 6, $P < 0.001$).

 © Cancer Research Campaign 1997

**Table 5** Association of patient and tumour factors with use of tamoxifen[a] in British Columbia and Ontario

| | British Columbia use of tamoxifen | | Ontario use of tamoxifen | | |
|---|---|---|---|---|---|
| | No. | % | No. | % | Interprovincial Mantel–Haenszel test for heterogeneity (*P*) |
| Overall rate of tamoxifen (+/– chemotherapy) | 163/932 | 17.5 | 259/911 | 28.4 | |
| Age (years) | | | | | |
| < 50 | 7/189 | 3.7 | 40/226 | 17.7 | |
| 50–65 | 67/318 | 21.1 | 115/346 | 33.2 | |
| > 65 | 89/425 | 20.9[b] | 104/339 | 30.7[b] | 0.028 |
| Median family income in area of residence | | | | | |
| < $35 000 | 24/122 | 19.7 | 24/121 | 19.8 | |
| $35 001–$50 000 | 103/570 | 18.1 | 129/484 | 26.7 | |
| > $50 000 | 36/240 | 15.0 | 106/306 | 34.6[c] | 0.011 |
| Tumour size | | | | | |
| < 2 cm | 74/577 | 12.8 | 134/534 | 25.1 | |
| 2–4 cm | 78/314 | 24.8 | 109/331 | 32.9 | |
| > 4 cm | 10/38 | 26.3 | 14/36 | 38.9 | |
| Unreported | 1/3 | 33.3[b] | 2/10 | 20.0[d] | 0.254 |
| Tumour grade | | | | | |
| Well | 14/103 | 13.6 | 23/99 | 23.2 | |
| Moderate | 63/388 | 16.2 | 97/303 | 32.0 | |
| Poor | 58/313 | 18.5 | 60/182 | 33.0 | |
| Unreported | 28/128 | 21.9 | 79/327 | 24.2[e] | 0.098 |
| LVN invasion | | | | | |
| Absent | 61/641 | 9.5 | 57/224 | 25.5 | |
| Present | 90/198 | 45.5 | 45/108 | 41.7 | |
| Unreported | 12/93 | 12.9[b] | 157/579 | 27.1[f] | < 0.0001 |
| Oestrogen receptor | | | | | |
| Positive | 127/565 | 22.5 | 213/585 | 36.4 | |
| Negative | 13/146 | 8.9 | 22/174 | 12.6 | |
| Unreported | 23/221 | 10.4[b] | 24/152 | 15.8[b] | 0.674 |

[a]Excludes cases on clinical trials of adjuvant systemic therapy or those in whom Tamoxifen treatment status was unknown. Intraprovincial chi square: [b]$P < 0.001$; [c]$P = 0.004$; [d]$P = 0.037$; [e]$P = 0.046$; [f]$P = 0.005$. All other categories $P > 0.2$

**Table 6** Proportion of patients who received adjuvant systemic therapy in each diagnostic grouping as per British Columbia guidelines

| | British Columbia | | | | | Ontario | | | | |
|---|---|---|---|---|---|---|---|---|---|---|
| | Total | No adjuvant therapy | Adjuvant chemotherapy | Tamoxifen | Tamoxifen and chemotherapy | Total | No adjuvant therapy | Adjuvant chemotherapy | Tamoxifen | Tamoxifen and chemotherapy |
| | *n* | % | % | % | % | *n* | % | % | % | % |
| < 50 years low risk | 119 | **84.9** | 12.6 | – | 2.5 | 167 | **70.1** | 12.0 | 14.4 | 3.6 |
| < 50 years high risk | 70 | 15.7 | **78.6** | 2.9 | 2.9 | 57 | 40.4 | **42.1** | 15.8 | 1.8 |
| 50–65 years low risk | 236 | **90.3** | 0.9 | 8.5 | 0.4 | 280 | **67.9** | 0.7 | 30.0 | 1.4 |
| 50–65 years high risk ER positive | 61 | 29.5 | 1.6 | **62.3** | **6.6** | 31 | 38.7 | – | **58.1** | **3.2** |
| 50–65 years high risk ER negative | 21 | 61.9 | **19.1** | 14.3 | 4.8 | 35 | 71.4 | **5.7** | 17.1 | 5.7 |
| > 65 years low risk | 327 | **85.9** | 0.3 | 13.8 | – | 289 | **69.6** | – | 30.1 | 0.4 |
| > 65 years high risk ER positive | 69 | 43.5 | – | **56.5** | – | 33 | 60.6 | – | **39.4** | – |
| > 65 years high risk ER negative | 28 | **82.1** | – | 17.9 | – | 16 | **81.3** | – | 18.8 | – |

Numbers in bold represent the treatment by the British Columbia guidelines.

### DISCUSSION

In this study, we observed greater consistency in the use of adjuvant systemic therapy in British Columbia than in Ontario, and the observed patterns in British Columbia corresponded to the British Columbia guidelines. As summarized in Table 7, higher consistency scores were observed in British Columbia regardless of the diagnostic grouping system used for analysis, and the effect persisted even in the assumption-free recursive partitioning procedure.

In three of the eight diagnostic groups defined by the British Columbia guidelines, recommended treatment was received by only 19.1–68.9% of women. Another report examines in detail the

Table 7 Consistency score[a] for different methods of diagnostic group development

| | Differences from British Columbia classification | British Columbia | Ontario | Z-statistic[b] |
|---|---|---|---|---|
| Group 1 | None | 0.758 | 0.514 | 9.36 |
| Group 2 | High risk, presence of 'poor' grade or presence of LVN invasion or tumour > 2cm if ER negative<br>If oestrogen receptors 'unknown' is assumed to be positive | 0.670 | 0.509 | 5.10 |
| Group 3 | High risk defined as in group 2<br>Risk is 'unknown' if oestrogen receptor is unknown. | 0.668 | 0.506 | 5.12 |
| Group 4 | High risk, presence of 'poor' grade or tumour > 2 cm if ER negative.<br>Presence or absence of LVN invasion not considered<br>If oestrogen receptor 'unknown' is assumed to be positive. | 0.657 | 0.513 | 4.39 |
| Group 5 | High risk is defined as in group 4<br>Risk is 'unknown' if oestrogen receptor unknown | 0.658 | 0.511 | 4.47 |
| Group 6 | Recursive partitioning model | 0.676 | 0.573 | 3.73 |

[a]If a grouping gives perfect classification, then the statistic is 1. If equal proportions of the patients in each group get the four treatments, that statistic is zero.
[b]For the comparison between the two provinces, all P-values are < 0.001.

reasons for less than optimal compliance with the British Columbia guidelines for these groups of women (Olivotto et al, 1997). In Ontario, however, only 5.7–61.3% of similar women received treatment as recommended by the British Columbia guidelines, supporting the suggestion that in British Columbia, treatment guidelines were incorporated into therapeutic decisions, at least by some physicians.

We also observed patterns of pathology reporting in British Columbia that suggest that pathologists were aware of the factors that clinicians were using for assessment of the risk of metastatic potential according to the British Columbia guidelines. This is most evident for reporting of LVN, in which a comment regarding LVN was made in 90% of cases. In contrast, in Ontario, where, in 1991, LVN was not commonly used as a prognostic factor, LVN was commented upon in only 36.3% of cases. Thus, it appears that the British Columbia guidelines led to pathology reports that included those factors relevant to the treatment decision. The standardized format for pathology reporting in the cancer centres may have increased awareness in the community pathology setting of these factors required for treatment recommendation.

We also observed a stronger correlation of both LVN status and age with recommendations for both chemotherapy and tamoxifen in British Columbia than in Ontario (Tables 4 and 5), supporting the view that physicians were aware of and using the British Columbia guidelines in British Columbia. The associations between all other independent variables and tamoxifen were not statistically different in the two provinces, with the exception of residence in areas of higher median family income for a recommendation for tamoxifen.

The observed trend toward increased use of adjuvant systemic therapy in women residing in areas of higher socioeconomic status in the Ontario cohort must be interpreted with caution as the variable represents median family income of the area of residence rather than actual family income of the cases. However, with these limitations in mind, there did appear to be a trend to greater use of tamoxifen in women residing in areas of higher socioeconomic status in Ontario. Whether this is due to differences in treatments offered or in patient expectations of treatment cannot be answered by this study. However, the lack of a similar trend in the British Columbia cohort suggests that provincial treatment guidelines may have played a role in limiting the influence of socioeconomic status on treatments recommended in that province.

The evaluation of the impact of treatment guidelines on patterns of care is problematic. Many of the studies published to date were not truly population based (McPhee et al, 1986; McCarthy and Bore, 1991; Studnicki et al, 1993; Grimshaw et al, 1995). Most studies did not compare practice patterns in centres with and without guidelines and therefore, even if guideline compliance was observed, it has not been possible to distinguish between true effects of treatment guidelines and general physician knowledge of good medical practice that may be adopted irrespective of the existence of treatment guidelines. Similarly, these studies have not been able to determine whether consistency of care, as measured by the homogeneity of treatments received, is improved by the presence of guidelines.

The strengths of the current study include the use of a population-based cohort rather than a selected population referred to cancer centres and the retrieval of data from actual source documents rather than solely from administrative data sets. Both of these aspects should improve the generalizability and validity of our results. Our approach also allows a comparison of consistency of care in the two provinces without making any assumptions about whether the British Columbia treatment guidelines are correct. Another strength is the comparison of patterns of care in two provinces that are similar in many respects. For example, British Columbia and Ontario both have populations of similar socioeconomic makeup (Statistics Canada, 1992) that reside largely in urban areas, universal health care insurance, similar medical training programmes and similar cancer care systems consisting of regional cancer centres that are responsible for the delivery of all radiation therapy.

Limitations of our study include possible undetected differences in data quality in the two provinces, some missing data on chemotherapy and tamoxifen use in Ontario, a lack of detailed information on co-morbidity that may have influenced treatment decisions and patient preferences for treatment. Although most of the tumour, patient and physician characteristics were comparable in the two provinces, the average age of the British Columbia cohort was somewhat older than the Ontario cohort. This may be explained partly by the older average age in British Columbia (Statistics Canada, 1992) and the higher incidence of breast cancer

 © Cancer Research Campaign 1997

in British Columbia (National Cancer Institute of Canada, 1995), but may also relate to differential selection of patients for axillary dissection. Even after stratification by age, however, differences in consistency of care persisted in the two provinces. Another limitation of our study is the inability to totally dissect the impact of treatment guidelines from small differences in the organization of the medical care systems. Although British Columbia and Ontario provide similar per capita cancer services, British Columbia, which had a 1991 population of 3.3 million, had one cancer care agency with two cancer centres, whereas Ontario, with a 1991 population of 10 million, had two cancer care organizations that operated nine regional cancer centres. In British Columbia, although there are no restrictions on drug prescriptions, the British Columbia Cancer Agency controls a provincial centralized formulary for anti-cancer drugs, and reimbursement occurs only after the patient is registered with, but not necessarily seen at, the British Columbia Cancer Agency. No centralized formulary for cancer drugs exists in Ontario. The extent to which these differences may have contributed to our observations is unknown.

Our study did not examine appropriateness or quality of care and is not intended to comment on these aspects in either province, nor is this an assessment of whether the guidelines in effect in British Columbia in 1991 were correct. Our results do, however, support the view that, once optimal adjuvant systemic therapy is defined, treatment guidelines can play a role in promoting consistent patterns of care and reducing the impact of extraneous factors such as socioeconomic status.

Although one goal of treatment guidelines is the timely dissemination of new information to treating physicians, paradoxically, strict adherence to treatment guidelines may actually impede the rapid introduction of newly reported therapies into clinical practice. For example, in this 1991 cohort, tamoxifen was used more commonly, albeit inconsistently, in Ontario than in British Columbia for women with smaller ER-positive tumours, and there has subsequently been an increasing tendency to prescribe tamoxifen in this setting (Glick et al, 1993). Thus, although guidelines appear to improve consistency of care, their role in improving quality of care is highly dependent on frequent review and incorporation of new research findings into the guidelines, as well as on appropriate and rapid dissemination of the updated guidelines (Mittman et al, 1992; Browman et al, 1995; Grimshaw et al, 1995).

### ACKNOWLEDGEMENTS

The assistance of Keyi Wu in statistical programming, Rebecca MacBride, Eileen Patchett and Bruna Rodrigues in preparation of the manuscript and the outstanding effort of Patricia Pinfold in overall project coordination is acknowledged. Supported by the National Cancer Institute of Canada through funds raised by the Canadian Cancer Society. Dr Goel is supported in part by a National Health Scholar Award from Health Canada.

### REFERENCES

Audet AM, Greenfield S and Field M (1990) Medical practice guidelines: current activities and future directions. *Ann Intern Med* **13**: 709–714

Breiman L, Friedman J, Olshen R, Stone CJ (1994) *Classification and Regression Trees*. Breiman L, Friedman J and Olshen R (eds). Wadsworth: Belmont

Browman GP, Levine MN, Mohide E, Hayward RS, Pritchard KI, Gafni A and Laupacis A (1995) The practice guidelines development cycle: a conceptual tool for practice guidelines development and implementation. *J Clin Oncol* **13**: 502–512

Canadian Hospital Association (1991) *Canadian Hospital Directory 1991–1992* Canadian Hospital Association: Ottawa

Canadian Medical Directory (37th edn) (1991) Southam Business Communications, Don Mills

Committee to Advise the Public Health Service on Clinical Practice Guidelines, Institute of Medicine (1990) Clinical practice guidelines: directions for a new program. *Natl Acad Pr* **38**: Washington

CRC Adjuvant Breast Trial Working Party (1988) Cyclophosphamide and tamoxifen as adjuvant therapies in the management of breast cancer. *Br J Cancer* **57**: 604–607

Early Breast Cancer Trialists' Collaborative Group (1992) Systemic treatment of early breast cancer by hormonal, cytotoxic or immune therapy: 133 randomized trials involving 31,000 recurrences and 24,000 deaths among 75,000 women. *Lancet* **339**: 1–5, 71–85

Fisher B, Redmond C, Fisher ER and Caplan R (1988) Relative worth of estrogen or progesterone receptor and pathologic characteristics of differentiation as indicators of prognosis in node negative breast cancer patients: findings from the National Surgical Adjuvant Breast and Bowel Project Protocol B-06. *J Clin Oncol* **6**: 1076–1087

Fisher B, Redmond C, Dimitrov NV, Bowman D, Legualt-Poisson S, Wickerham DL, Wolmark N, Fisher ER, Margolese R, Sutherland C, Glass A, Foster R, Caplan R, Abramson N, Allegra JC, Beazley R, Cruz AB, Elias GE, Feldman MI, Henderson C, Levick SH, Oishi R, Poisson R, Robidoux A, Rosen R, Shah NM, Shibata H, State D, Terz J, Welling R and Wozniak T (1989*a*) A randomized clinical trial evaluating sequential methotrexate and fluorouracil in the treatment of patients with node-negative breast cancer who have estrogen-receptor-negative tumors. *N Engl J Med* **320**: 473–478

Fisher B, Costantino J, Redmond C, Poisson R, Bowman D, Couture J, Dimitrov NV, Wolmark N, Wickerham DL, Fisher ER, Margolese R, Robidoux A, Shibata H, Terz J, Paterson AHG, Feldman MI, Farrar W, Evans J, Lickely L, Ketner M, Abramson N, Allegra JC, Beazley R, Berry J, Boatman KK, Bowman D, Carmichael D, Cruz AB, Davies R, Deckers P, Desser R, Economou S, Elias EG, Foster RS, Frazier TG, Glass AG, Jochimsen P, Jubelirer S, Kardinal CG, Keyserlingk JR, Lerner HJ, Levick SN, Mahoney L, Mowry P, Nicola M, Oishi R, Pasquale D, Perrault D, Peters G, Pugh R, Robert N, Robidoux A, Shibata H, Spector J, Sponzo R, Sterchi JM, Sutherland CM, Terz J, Thiessen R, Thirlwell M, Welling R, Wold H and Wozniak T (1989*b*) A randomized clinical trial evaluating tamoxifen in the treatment of patients with node-negative breast cancer who have estrogen-receptor-positive tumors. *N Engl J Med* **320**: 479–484

Ford LG, Hunter CP, Diehr P, Frelick RW and Yates J (1987) Effects of patient management guidelines on physician practice patterns: the Community Hospital Oncology Program experience. *J Clin Oncol* **5**: 504–511

Glick J, Gelber R, Goldhirsch A and Senn HJ (1993) Adjuvant therapy of primary breast cancer: closing summary. In: *Adjuvant Therapy of Breast Cancer IV*, Senn HJ, Gelber R, Goldhirsch A and Thurlimann B (eds), pp. 289–300. Springer: Berlin

Gregorio DI, Strelez LA and Sporn JA (1990) Attitudes and practices regarding adjuvant chemotherapy in node-negative breast cancer. *J Cancer Educ* **5**: 187–192

Grilli R, Apolone G, Marsoni S, Nicolucci A, Zola P and Liberati A (1991) The impact of patient management guidelines on the care of breast, colorectal, and ovarian cancer patients in Italy. *Medical Care* **29**: 50–63

Grimshaw J, Freemantle N, Wallace S, Russell I, Hurwitz B, Watt I, Long A and Sheldon T (1995) Developing and implementing clinical practice guidelines. *Quality in Health Care* **4**: 5–64

Henderson IC (1991) Adjuvant systemic therapy of early breast cancer. In *Breast Diseases*, 2nd edn, Harris J, Hellman S, Henderson IC and Kinne D (eds), pp. 427–486. JB Lippincott: Philadelphia.

Johnson TP, Ford L, Warnecke RB, Nayfield SG, Kaluzny A, Cutter G, Gillings D, Sondik E and Ozer H (1994) Effect of a National Cancer Institute Clinical Alert on breast cancer practice patterns. *J Clin Oncol* **12**: 1783–1788

Kanouse DE and Jacoby I (1988) When does information change practitioners' behavior? *Intl J Technol Assess Health Care* **4**: 27–33

Kelly JT and Toepp MC (1992) Practice parameters: development, evaluation, dissemination, and implementation. *Q Rev Bull* **18**: 405–409

Ludwig Breast Cancer Study Group (1989) Prolonged disease-free survival after one course of perioperative adjuvant chemotherapy for node-negative breast cancer. *N Engl J Med* **320**: 491–496

McBride M and Donaldson L (1987) Ascertainment and data collection for a cancer registry. *British Columbia Med J* **29**: 30–40

McCarthy M and Bore J (1991) Treatment of breast cancer in two teaching hospitals: a comparison with consensus guidelines. *Eur J Cancer* **5**: 579–582

© Cancer Research Campaign 1997

McGuire WL (1989) Adjuvant therapy of node-negative breast cancer (editorial). *N Engl J Med* **320**: 525–527

McGuire W and Clark G (1992) Prognostic factors and treatment decisions in axillary-node-negative breast cancer. *N Engl J Med* **326**: 1756–1761

McPhee S, Richard R and Solkowitz S (1986) Performance of cancer screening in a university general internal medicine practice: comparison with the American Cancer Society guidelines. *J Gen Intern Med* **1**: 275–281

Mansour EG, Gray R, Shatila AH, Osbourne CK, Tormey DC, Gilchrist KW, Cooper MR and Falkson G (1989) Efficacy of adjuvant chemotherapy in high-risk node-negative breast cancer: an Intergroup study. *N Engl J Med* **320**: 485–490

Mittman B and Siu A (1992) Changing provider behavior: applying research on outcomes and effectiveness in health care. In *Improving Health Policy and Management*. Shortell S and Reinhardt U (eds), pp. 95–226. Health Administration Press: Ann Arbor

National Cancer Institute of Canada (1995) *Canadian Cancer Statistics* 1995. National Cancer Institute of Canada, Toronto.

Olivotto I, Bajdik C, Plenderleith I, Coppin CM, Gelmon KA, Jackson SM, Ragaz J, Wilson KS and Worth A (1994) Adjuvant systemic therapy and survival from breast cancer. *N Engl J Med* **330**: 805–810

Olivotto IA, Coldman AJ, Hislop TG, Trevisan CH, Goel V and Sawka C (1997) Compliance with practice guidelines for node-negative breast cancer. *J Clin Oncol* **15**: 216–222

Robles SC, Marrett LD, Clarke EA and Risch HA (1988) An application of capture–recapture methods to the estimation of completeness of cancer registration. *J Clin Epidemiol* **41**: 495–501

Scottish Cancer Trials Office (1987) Adjuvant tamoxifen in the management of operable breast cancer. *Lancet* **2**: 171–175

Shapiro DW, Lasker RD, Bindman AB and Lee PR (1993) Containing costs while improving quality of care: the role of profiling and practice guidelines. *Annu Rev Publ Health* **14**: 219–241

Statistics Canada (1992) *1991 Census of Canada*. Supply and Services Canada: Ottawa.

Studnicki J, Schapira DV, Bradham DD, Clark RA and Jarrett A (1993) Response to the National Cancer Institute Clinical Alert. The effect of practice guidelines on two hospitals in the same medical community. *Cancer* **72**: 2986–2992

Wilkins R (1993) Use of postal codes and analysis of health data. *Health Reports* **5**: 157–177

### APPENDIX 1: CALCULATION OF THE CONSISTENCY SCORE AND ITS SIGNIFICANCE

A consistency score was developed to assess the homogeneity of treatments across diagnostic strata. For each diagnostic grouping the proportion of cases receiving the most frequently used treatment was calculated. These quantities were summed across all the strata within a province, weighting by the proportion of subjects in that stratum in each province. The resulting quantity was then transformed to yield a consistency score that could range between zero and one in each province. The consistency score ($C$) is calculated according to the following formula:

$$C = \frac{t}{(t-1)}\left[\sum_{i=1}^{k} w_i P_i\right] - \frac{1}{(t-1)}$$

where $k$ is the number of diagnostic strata, $t$ the number of possible treatments, $p_i$ is the proportion of subjects within a strata receiving the most frequently used treatment and $w_i$ is a weighting factor that sums to 1. We used the proportion of subjects in each strata as the weighting factor.

A test for comparing consistency scores was constructed by considering the number receiving the most frequent treatment as a binary variable in each treatment stratum. It was assumed that the underlying frequency of the most commonly given treatment was sufficiently greater than the underlying frequency of the other treatments so that the distribution of the observed maximum was well approximated by a binomial variable in each stratum. A significance test for differences in the statistic $C$ between the provinces was constructed using its approximate asymptomatic normality where the variance was calculated in the usual way for a binomial distribution.

 © Cancer Research Campaign 1997